# Profiling Protein Post-Translational Modifications in Plasma-derived Extracellular Vesicles as Fingerprints for Breast Cancer Subtypes

**DOI:** 10.21203/rs.3.rs-7294729/v1

**Published:** 2025-08-21

**Authors:** Marco Hadisurya, Hillary Andaluz Aguilar, I-Hsuan Chen, Mengting Xu, J. Sebastian Paez, Rachit Bisht, Jyoti Singh, Zheng-Chi Lee, Amirhesam Mashaollahi, Anton B Iliuk, Weizhou Zhang, W. Andy Tao

**Affiliations:** Purdue University; Purdue University; Purdue University; Purdue University; Purdue University; Purdue University; Purdue University; Purdue University; Purdue University; Tymora Analytical Operations; University of Iowa; Purdue University

## Abstract

Addressing tumor heterogeneity in breast cancer (BC) research is crucial, given the distinct subtypes like triple-negative (TN), luminal A/B (LAB), and HER2, requiring precise differentiation for effective treatment. This study introduces a non-invasive method by analyzing post-translationally modified proteins in plasma extracellular vesicles (EVs), which play a role in immune regulation and intercellular communication. Examining modifications like phosphorylation, acetylation and glycosylation in EVs provides insights into BC dynamics. One hundred and one plasma samples from LAB BC, TN BC and healthy individuals underwent discovery and validation experiments. The study identified over 28,000 unique non-modified peptides, 5,000 phosphopeptides, 680 acetyl peptides and 1,300 glycopeptides that were successfully characterized. Bioinformatics analyses revealed significant overexpression of 815 non-modified proteins, 3,958 phosphopeptides, 352 acetyl peptides and 895 glycopeptides in LAB BC or TN BC subtypes. Phosphorylated and glycosylated PD-L1 peptides emerged as potential markers for BC, regardless of subtype. Aligning the findings with literature and PAM50 gene signatures highlighted markers correlated with lower survival rates. The study also conducted 123 scheduled parallel reaction monitoring (PRM) analyses, leveraging machine learning to pinpoint a panel of specific modification sites with high accuracy for subtype differentiation. This research reveals diagnostic markers and enhances understanding of the molecular landscape, contributing to more effective and personalized BC diagnostics and treatments.

## INTRODUCTION

Extracellular vesicles (EVs), including exosomes and microvesicles, contain a wealth of nucleic acids, proteins and signaling molecules vital for intercellular communication [1]. With growing evidence that EVs reflect the molecular signature of the parent cell, the increase in the understanding of EV compositions is critical for their establishment as valuable repertoires of biomarkers for human diseases [2]. In the context of cancer, this opens the door to applying EV-based liquid biopsy for early detection, ahead of imaging-based diagnoses that are currently prevailing. Research into potential biomarkers isolated from EVs has been propelled by the developed methods and tools to acquire them minimally and non-invasively, reinforcing their great diagnostic potential.

Protein post-translational modifications (PTMs), including phosphorylation, glycosylation and acetylation, play crucial roles in various essential cellular processes and serve as key regulatory mechanisms for cellular physiological functions. These modifications are significant in the development of effective and targeted therapies [3]. Discovery and profiling of PTMs have established their relevance in EVs and associated EV functions and novel applications. Tandem mass spectrometry (MS/MS) has been the main tool for examining PTMs on a global proteomic level [4]. Several studies, including some from our lab, have previously demonstrated MS-based methods to profile phosphoproteome and glycoproteome in EVs from biofluids such as plasma and urine [5–11]. These studies assert the comprehensive PTM proteomics approach as a viable path towards developing non-invasive diagnostic tools, as each different pathology will have specific and dynamic molecular signatures carried by EVs.

This study presents a comprehensive profiling analysis of protein PTMs in EVs to distinguish breast cancer (BC) subtypes. BC has been thoroughly characterized, and its molecular complexity and heterogeneity are well established, with distinct molecular subtypes influencing clinical outcomes [12]. Therefore, a potential diagnostic modality would be greatly beneficial if it could distinguish between molecular subtypes. These subtypes, identified by the presence of estrogen receptor (ER), progesterone receptor (PR), or the epidermal growth factor receptor (ERBB2/HER2), are categorized into three primary groups: luminal A/B (LAB), HER2-positive, and triple-negative (TN) [13]. The subtype luminal A has the ER expression, the highest expression of PR receptor genes, and the lowest expression of proliferation-related genes [14]. The subtype luminal B is characterized by the expression of ER, lower expression of PR receptor genes [15], and high expression of proliferation-related genes [16]. Importantly, some luminal B subtypes can also be HER2-positive. However, despite having the best clinical outcome and survival rate, most deaths from metastatic BC come from patients with LAB subtypes due to their high incidence.

In the case of LAB subtypes, characterized by the expression of ER and/or PR, endocrine therapies such as tamoxifen or aromatase inhibitors are the primary treatments. These therapies target hormone receptors to inhibit tumor growth. When combined with other treatments, like ovarian suppression in premenopausal women or chemotherapy in high-risk cases, endocrine therapies are highly effective for LAB BC. Although endocrine therapy remains the main treatment, it can lose effectiveness due to primary or acquired endocrine resistance [17]. Endocrine therapies for ER-positive BC include tamoxifen, which has long been considered the ‘gold standard’ and is one of the oldest treatments. Other endocrine therapies include ovarian suppression agents like Lupron (leuprolide) and Zoladex (goserelin), as well as aromatase inhibitors like Anastrozole (Arimidex) and Letrozole (Femara). On the other hand, the TN BC subtype, which lacks the expression of ER, PR, and HER2, presents a particularly poor prognosis due to the limited treatment options available and its aggressive nature.

Additionally, the recurrence rate of BC is high due to either primary or acquired resistance, or insufficient pharmacological treatment, leading to a significant number of therapeutic or preventive mastectomies [18]. It has been found that disease markers based on EVs can be detected before the disease develops or can serve to help identify BC relapse [10, 19]. Early identification of subtypes in the initial stages of BC would enable patients to receive appropriate therapies sooner, improving survival rates. Furthermore, assessing plasma EVs in patients who have undergone mastectomies could be advantageous since they lack breast tissue, making this approach particularly useful.

Combining multiple PTM signatures has already been demonstrated as an effective strategy to map lung cancer signaling networks [20]. Previous research has also demonstrated that profiling BC using an omics-style approach can differentiate between molecular subtypes [19, 21]. However, all prior multi-PTM proteome-wide studies have been conducted using either cell culture systems or tissue samples. We propose that integrating PTM biosignatures from plasma EVs would significantly improve the detection and prognosis of malignancies, specifically for clinical profiling BC subtypes. Analyzing proteins with PTMs from EV cargo provides insight into cellular regulation and signaling pathways that reflect disease status. The combination of different PTMs isolated from plasma EVs in BC patients could enable the clear differentiation of BC subtypes, offering a novel approach to monitoring patient pathophysiology. In this study, we extend our analytical pipeline to examine EV protein PTMs from clinical plasma samples, creating biosignatures to distinguish BC subtypes and assist in treatment selection. Our research particularly focuses on LAB and TN subtypes due to their unique profiles, with luminal B being predominantly metastatic and TN lacking effective targeted pharmacological treatments.

In this study, we present a novel pipeline for the sequential enrichment and quantitative analysis of phosphorylated, N-glycosylated, and acetylated peptides from plasma-derived EVs, enabling comprehensive proteome and PTMome characterization within the same biological sample. By applying this approach to plasma samples from healthy individuals and patients with LAB and TN BC subtypes, we identified subtype-specific EV protein modifications with high reproducibility and substantial overlap with EV databases, underscoring the robustness of the method. Our findings reveal that PTM profiling provides significantly greater discriminatory power than proteome analysis alone, identifying critical pathways such as EGFR, MAPK, and Rho GTPase signaling in TN BC, and dysregulated G2/M phases in LAB subtypes. BC PTM markers like phosphorylated and glycosylated PD-L1 and the phosphorylation site of ENO1 at serine 419 further highlight their potential as diagnostic and therapeutic targets. Through Reactome enrichment analyses, this integrative PTM platform offers novel insights into BC subtype biology, emphasizing the critical role of PTMs in distinguishing subtypes and guiding treatment decisions. Overall, this plasma-derived EV pipeline demonstrates its potential as a viable alternative for identifying clinically relevant targets in breast cancer pathology and treatment resistance.

## METHODS

### Plasma samples

The Iowa University Institutional Review Board approved the use of human plasma samples. For the global PTMome experiment, blood samples were collected from healthy females through the Susan G. Komen Tissue Bank, while samples from BC subtypes were obtained through the University of Iowa Biobank (**See Supplementary Data 1 for the complete patient manifest**). Plasma samples were processed according to a standard protocol. Specifically, processing began within 30 minutes of blood being drawn into a tube containing ethylenediaminetetraacetic acid (EDTA). The samples were then centrifuged at 3500 rpm for 30 minutes to remove cell debris and platelets. After processing, the samples were stored at −80°C until they were needed for further use.

### Extracellular vesicle isolation

EV isolation was carried out following the established protocol [9]. For the global PTM experiment, 5 ml pooled plasma samples were collected from both healthy individuals and BC patients, serving as technical replicates. The plasma samples were first centrifuged at 20,000 × g at 4°C for 1 hour. The resulting pellets, which were labeled as large EVs (primarily microvesicles, were washed with cold PBS and centrifuged again at 20,000 × g at 4°C for another hour. The supernatant from the initial centrifugation was then subjected to a second centrifugation at 100,000 × g at 4°C for 1 hour. The resulting pellets, which were labeled as small EVs (primarily exosomes, were also washed with cold PBS and centrifuged at 100,000 × g for an additional hour. Finally, the two separately isolated EV fractions were combined during the sample lysis process.

### Enzymatic digestion

Enzymatic digestion was carried out using phase transfer surfactant-aided (PTS) digestion [22]. EVs were dissolved in a lysis buffer containing 12mM sodium deoxycholate (SDC), 12mM sodium lauroyl sarcosinate (SLS), and a phosphatase inhibitor cocktail in 100mM Tris-HCl, pH 8.5. The proteins were reduced and alkylated using 10 mM tris-(2-carboxyethyl)phosphine (TCEP) and 40 mM chloroacetamide (CAA) at 95°C for 5 minutes. After alkylation, the proteins were diluted fivefold with 50mM triethylammonium bicarbonate (TEAB) and digested with Lys-C at an enzyme-to-protein ratio of 1:100 (w/w) for 3 hours at 37°C. Trypsin was then added at a final enzyme-to-protein ratio of 1:50 (w/w) for overnight digestion at 37°C. The resulting digested peptides were acidified with trifluoroacetic acid (TFA) to a final concentration of 0.5% TFA, and 250 μl of ethyl acetate was added to the 250 μl digested solution. The mixture was shaken for 2 minutes and centrifuged at 13,200 rpm for 2 minutes to separate the aqueous and organic phases. The aqueous phase was collected and desalted using a 100 mg Sep-Pak C18 column.

### Tyrosine phosphopeptides enrichment

Desalted peptides were resuspended in 50 mM Tris-HCl pH 7.5 and incubated with 20 uL of PT66 beads with rotation overnight at 4°C. The PT66 beads were washed sequentially with lysis buffer (50 mM Tris-HCl, 50 mM NaCl, 1% NP40 pH7.5) and water three times each for 10 minutes using end-over-end rotation to wash off the non-specific binding. Tyrosine phosphopeptides were sequentially eluted twice with 0.1%TFA and once with 0.1% TFA/50% ACN. The eluents were combined and dried under a vacuum.

### Lysine acetylation peptide enrichment

Immunoaffinity enrichment of lysine-acetylated peptides from EVs was performed according to the manufacturer’s instructions (PTMScan). In brief, 20ul of lysine acetylation antibody-conjugated beads were washed extensively with PBS. The flow-through from tyrosine phosphopeptides was mixed with lysine acetylation antibody beads and incubated for 2 hours at 4°C. The beads were washed twice with IAP buffer (50 mM MOPS, pH 7.2, 10 mM sodium phosphate, 50 mM NaCl) and three times with water. Peptides were eluted from beads with 0.15% TFA (sequential elutions of 55 μl followed by 50 μl, 10 minutes each elution at room temperature). The resulting peptides were desalted by SDB-XC stage tip and eluted with 40% ACN in 0.1% TFA. Eluted peptides were dried under a vacuum. The flow-through was desalted by SDB-XC stage tip and dried under vacuum.

### PolyMAC phosphopeptides enrichment

The flowthrough peptides after acetylation enrichment were enriched for phosphopeptides using PolyMAC-Ti silica beads according to the manufacturer’s instructions (Tymora Analytical Operations) [23]. The phosphopeptide eluates were collected and dried under a vacuum. The flow-through peptides were desalted by SDB-XC stage tip, and the eluent was dried for glycopeptide enrichment.

### N-Glycopeptides enrichment

The glycopeptide enrichment was performed according to the reported protocol. Desalted peptides were oxidized with 10 mM sodium periodate in 50% ACN and 0.1% TFA at room temperature and shaken in the dark for 30 minutes. Excess sodium periodate was quenched using 50 mM sodium sulfite for 15 minutes at room temperature and shaken in the dark. The samples were mixed with 50 μL hydrazide magnetic beads for individual and 100 μL for pooled samples and incubated with vigorous shaking at room temperature overnight for the coupling reaction. The magnetic beads were washed sequentially with 400 μL or 800 μL of 50% ACN, 0.1% TFA, and 1.5 M NaCl for individual and pooled samples, respectively, three times per solution for 1 minute per wash to remove non-coupled peptides. The beads were rinsed once with 100 μL or 200 μL of 1x GlycoBuffer 2 (NEB) for individual and pooled samples, respectively, and incubated with 3 μL or 4 μL of PNGase F (NEB) in 100 μL or 200 μL for individual and pooled samples respectively to cleave the N-glycans. After desalting, the released former N-glycopeptides were analyzed by liquid chromatography-tandem mass spectrometry (LC-MS/MS).

### LC-MS/MS

The PTM peptides were dissolved in 4 μL of 0.3% formic acid (FA) with 3% acetonitrile (ACN) and injected into an Easy-nLC 1200 system (Thermo Fisher Scientific). The peptides were separated using a 45 cm in-house packed column (360 μm outer diameter × 75 μm inner diameter) filled with C18 resin (2.2 μm, 100Å, Michrom Bioresources) and maintained at 50°C using a 30 cm column heater (Analytical Sales and Services). The mobile phase buffer consisted of 0.1% FA in ultra-pure water (buffer A), with an eluting buffer of 0.1% FA in 80% ACN (buffer B), run over a 45 or 60-minute linear gradient from 5–25% buffer B at a flow rate of 300 nL/minute. The Easy-nLC 1200 was directly interfaced with a high-resolution LTQ-Orbitrap Velos Pro mass spectrometer (Thermo Fisher Scientific), which operated in data-dependent mode. The mass spectrometer performed a full MS scan (m/z range of 350–1500, resolution of 30,000 at m/z 400) followed by MS/MS on the 10 most intense ions, with parameters set to 30% normalized collision energy, automatic gain control (AGC) of 3E4, and a maximum injection time of 100 ms. For parallel reaction monitoring (PRM), The Easy-nLC 1200 was coupled online with a Thermo Scientific^™^ Orbitrap Fusion^™^ Tribrid^™^ mass spectrometer, which was operated in data-dependent mode. The peptides in the inclusion list were selected for high-energy collisional dissociation (HCD) fragmentation (with a normalized collision energy (NCE) of 30%, AGC of 3e4, and a maximum injection time of 100 ms) for each full MS scan (covering m/z 350–1500 with a resolution of 120,000 at m/z 200).

### Data processing

The raw files were searched directly by UniProtKB database with no redundant entries using Proteome Discoverer software (version 2.5.0.400) with the Sequest HT and Byonic search engines. The initial precursor mass tolerance set at 10 ppm, fragment mass tolerance at 0.6 Da, minimum peak count as 1, PSM confidence FDR of 0.01 as strict and 0.05 as relaxed, maximum RT shift as 10 min, with hypothesis test of t-test (background based), protein summed abundance calculation, 100 as the maximum allowed fold change, and site probability threshold of 75. Search criteria included a static carbamidomethylation of cysteines (+ 57.0214 Da) and variable modifications of (1) oxidation (+ 15.9949 Da) on methionine residues, (2) acetylation (+ 42.011 Da) at N-terminus of protein, (3) phosphorylation(+ 79.996 Da) on serine, threonine or tyrosine residues for phosphorylation, (4) acetylation (+ 42.011 Da) on lysine residue, and (5) deamidation (+ 0.984 Da) on asparagine residues for glycosylation. The search was performed with full tryptic digestion and a maximum of two missed cleavages. The minimum and maximum peptide lengths were 6 and 144 amino acids, respectively. The glycosylation sites were further selected based on matching the N-X-S/T (X not Pro) motif.

### Quantitative data analysis

Data from the pooled and PRM experiments was analyzed using the Perseus software (version 1.6.15.0) and Skyline, respectively [24, 25]. For the quantification of both proteomic and PTM-omic datasets, the intensities of proteins and PTM sites were derived from Proteome Discoverer, and the missing values of intensities were replaced by a normal distribution with a downshift of 1.8 standard deviations and a width of 0.3 standard deviations. The significantly increased PTM sites or proteins in patient samples were identified by ANOVA multi-test with a permutation-based FDR cut-off of 0.05 for all data sets. The changed sites or proteins were used for the heatmap, and the imputed data set was normalized by z-score within each dataset. Moreover, the survival analysis was done using the GENT2 platform [26]. The Reactome and biological process enrichment analyses were performed using the ClusterProfiler package [27] in R language and environment for statistical computing (R Core Team, 2019) [28]. For the PRM data, after differential intensities at the modification site level were selected, the corresponding precursor peptides were selected and imported to Skyline.

### Machine Learning Approach

In this study, we aimed to identify targets that could differentiate between BC subtypes, focusing specifically on LAB, TN, and general BC. We utilized the peptides enriched for PTMs to identify the most significant targets. The validation dataset consisted of 41 samples, which included 19 LAB samples, 14 TN samples, and 8 control (normal) samples. We began with 111 potential targets identified from the initial data analysis. A two-step feature selection process was employed to refine this set. First, backward elimination reduced the target count to 30 by removing features with minimal impact on classification. The backward elimination was followed by exhaustive feature selection, further narrowing the list to a final set of 7 targets for each model. Feature selection was optimized using 5-fold cross-validation, dividing the entire training data into five subsets to maximize model performance.

Separate binary classification models were developed to address the complexity of differentiating subtypes. The first model distinguished LAB samples from TN and control samples, while the second model differentiated TN samples from LAB and control samples. All data processing, feature selection, and model training were conducted using Python with some of the following libraries: pandas 1.4.3 [29], numpy 1.20.1 [30], scikit-learn 1.1.1 [30] and mlxtend 0.20.0 [31] for extended feature selection utilities. Visualization of selected targets and model performance was accomplished using matplotlib 3.3.4 [32] and seaborn 0.11.2 [33], providing detailed plotting for metrics such as ROC and Variable Importance in Projection (VIP) plots.

Following feature selection, hyperparameter tuning was conducted to optimize model performance. We employed a two-stage process: a randomized search over 120 different combinations of hyperparameters, followed by an exhaustive grid search focused on the optimal combination sampled in the first stage. This process ensured the model was tuned for maximum performance. The best-performing hyperparameters were identified as follows: bootstrap set to True, max_depth set to 4, max_features set to ‘sqrt,’ min_samples_leaf set to 3, min_samples_split set to 2, and n_estimators set to 300. Once the optimal hyperparameters were selected, we trained the Random Forest Classifier on the refined parameter set 5 times and used cross-validation each time to ensure the accuracy of the classifier was consistent.

For the LAB-specific model, the peptide Q14517|FAT1_Glyco_TGALTVQNTTQLR was identified as the most significant feature, enhancing the ability to distinguish LAB cases from other samples. Meanwhile, Q06187|BTK_Phospho_STGDPQGVIR emerged as the key feature differentiating TN samples. The performance of each model was evaluated using Receiver Operating Characteristic (ROC) curves.

## RESULTS

### Sequential isolation and profiling of plasma-derived EV proteome and PTMome

**Figure 1.**
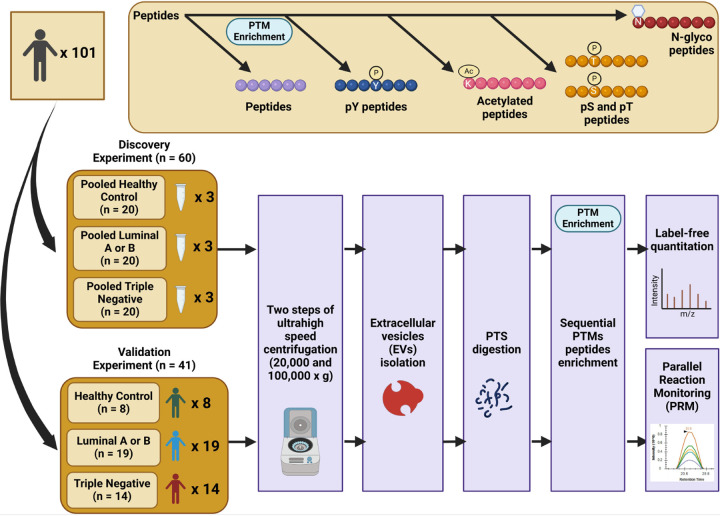
Workflow of the PTM-omics pipeline for plasma-derived EVs in healthy control and breast cancer patients. Plasma samples were collected and pooled from healthy individuals (n=20), patients with luminal A/B breast cancer (n=20), and patients with triple-negative breast cancer (n=20). Extracellular vesicles (EVs) were isolated from the plasma using a series of high-speed and ultra-high-speed centrifugation steps. Following isolation, EVs were lysed to extract proteins, which were then enzymatically digested with LysC and trypsin. To enrich for specific post-translational modifications (PTMs), a sequential enrichment protocol was used: tyrosine-phosphorylated peptides were isolated using the PT66 antibody, lysine-acetylated peptides were enriched next, followed by serine/threonine-phosphorylated peptides via PolyMAC, and finally, glycopeptides were captured using hydrazide chemistry. The enriched samples were analyzed by LC-MS/MS using a high-speed, high-resolution mass spectrometer with technical replicates. Label-free quantitation was employed to identify differentially modified proteins in plasma from control and breast cancer subtypes. Potential PTM targets were then validated by targeted proteomics (PRM) in a set of 41 individual plasma samples.

**Figure 2.**
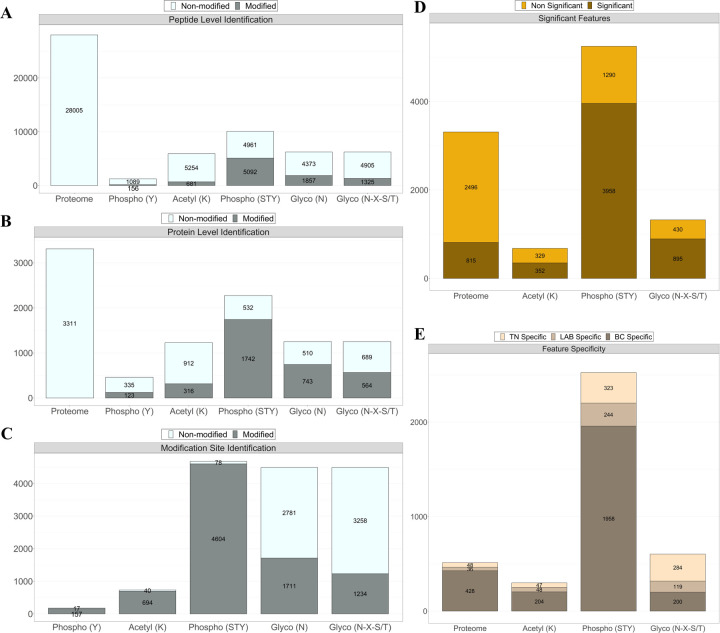
Identified features of proteins and PTM-modified peptides across healthy control, luminal A/B, and triple-negative breast cancer samples. Shown are the number of identifications at the peptide level (A), protein level (B), and modification site level (C) across different PTMs. (D) Differentially modified proteins and peptides, highlighting significant features as visualized in Figure 3A. (E) Feature specificity analysis, detailing unique, subtype-specific proteins and PTMs that distinguish breast cancer subtypes.

We have recently proposed a pipeline to sequentially analyze phospho- and N-glyco-proteomics of EVs from the same plasma samples [9]. Here, we expanded the workflow for isolation of EVs from plasma, sequential enrichment of phosphorylated, N-glycosylated, and acetylated peptides for quantitative proteomic analyses (Fig. 1). Human plasma was centrifuged first at low speed to remove cell debris, and EVs were isolated through high-speed (20k × g) and ultra-high-speed centrifugations (100k × g), an approach that has been used in previous studies [9, 10]. For the initial screening (discovery experiment), plasma samples were collected and pooled from healthy individuals (n = 20) and from patients diagnosed with LAB BC (n = 20) and TN BC (n = 20). Each pool had a final volume of 5 mL for three technical replicates and consisted of 0.25 mL taken from each patient. After lysis of EVs, proteins were extracted, denatured, alkylated, and enzymatically digested using Lys-C and trypsin with the aid of phase-transfer surfactants for fewer missed tryptic sites and better digestion [22]. Sequential enrichment was performed for each sample, starting with tyrosine phosphorylation using PT66 antibody, followed by lysine acetylation, S/T phosphorylation by PolyMAC [23], and N-glycopeptide enrichment using hydrazide chemistry approach [34]. Label-free quantitation was performed by LC-MS/MS on a high-speed and high-resolution mass spectrometer to determine the differential PTM proteins in plasma-derived EVs from control and BC subtype patients. Based on a pipeline that allowed for the enrichment of three PTMs from the same clinical samples, the platform enabled us to identify 28,005 unique non-modified peptides, 5,248 phosphopeptides (STY), 1,325 glycopeptides (N-X-S/T), and 681 acetylated peptides, representing 3,311 proteins, 1,865 phosphoproteins, 564 N-glycoproteins, and 316 acetylated proteins, with 1% FDR cut-off (Fig. 2A–E, **see Supplementary Data 2 for the complete data**). We overlapped our EV proteome and PTMome data against an EV-curated database extracted from Vesiclepedia and ExoCarta [35, 36]. Results showed an 84% overlap between our identified proteins and the EV database, indicating overall selective and efficient isolation and identification of EV proteins (**Supplementary Fig. 1, see Supplementary Data 3 for table format**). We carried out Pearson correlation to examine the reproducibility of the approach. As shown in **Supplementary Fig. 2** (**see Supplementary Data 4 for input files**), the reproducibility of the EVs isolation and PTM analysis across different samples is relatively high, with Pearson correlations between 0.71–0.98. Moreover, the coefficient of variation across different samples is below 2% (**Supplementary Fig. 3, see Supplementary Data 5 for table format**).

**Figure 3.**
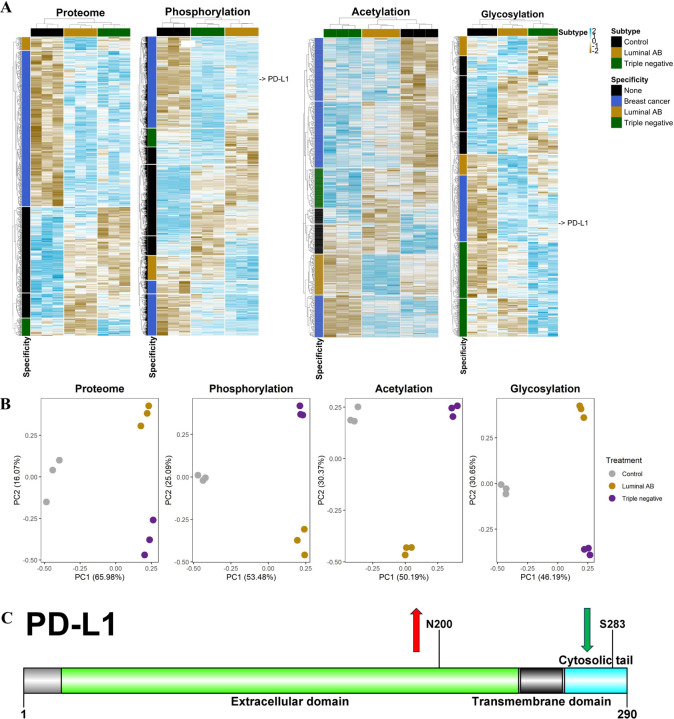
Label-free quantitative analysis of EV proteome, phosphopeptidome, acetyl peptidome, and N-glyco peptidome comparing healthy controls and breast cancer subtypes. (A) Quantitative analysis of each modified peptidome in LAB and TNBC samples relative to controls. We assigned those who are upregulated in LAB as LAB specific, TN as TN specific, and both LAB and TN as BC specific. (B) Principal component analysis (PCA) shows that replicates for modified peptidomes cluster more tightly, highlighting that PTMs improve differentiation between breast cancer subtypes. (C) The glycosylation at N200 and phosphorylation at S283 on PDL1 were upregulated and downregulated, respectively.

Label-free quantitative analysis of EV proteome, phosphopeptidome, acetyl peptidome, and N-glyco peptidome between healthy controls and BC subtypes is illustrated in **Fig. 3A (see Supplementary Data 2 for table format)**, where we assigned those features that are upregulated in LAB as LAB-specific, TN as TN-specific, and in both LAB and TN as BC-specific. Although quantitative analyses of EV proteomes reveal some features that differentiate between LAB BC and TN BC compared to controls, better distinctions across subtypes can be made from the phosphopeptidome, acetyl peptidome (K), and N-glyco peptidome data. This supports the claim that proteomics analysis alone might not provide adequate information about the differentiation among BC subtypes. The data also reveal that the PTM differences among BC subtypes and controls are not merely a result of differences in protein expression, therefore justifying the need to integrate multiple PTM analyses for specific signaling and regulation events with BC patients. As a notable example, programmed death-ligand 1 (PD-L1) was identified in EV phosphopeptidome and N-glyco peptidome, with N-glyco being significantly upregulated and phospho significantly downregulated in TB BC patients compared to controls (Fig. 3C). PD-L1 has been reported as significantly expressed in cancer cells and breast cancer specifically, especially in TN BC patients, according to several studies [37–41]. Recently, a monoclonal antibody targeting the glycosylated PD-L1 form was found to inhibit PD-L1/PD-1 interaction [41]. This manipulation produced a novel response in which targeted and adjacent TN BC cancer cells were killed via internalization and degradation of the PD-L1/PD-1 complex [41]. Furthermore, this monoclonal antibody also induced a bystander-killing effect on adjacent TN BC cells lacking PD-L1 expression without detectable toxicity, demonstrating the therapeutic potential of targeting protein glycosylation [41]. This evidence further reinforces the importance of analyzing glycosylation events in disease models, which is highly emphasized in our methodological approach.

We also performed principal component analysis (PCA) on each modification (Fig. 3B, **see Supplementary Data 6 for input files**). The PCA plot illustrated the relationships and similarities among sample replicates and experimental conditions, aiming to simplify the complexity of a dataset with numerous variables by projecting it into a lower-dimensional space. Interestingly, the three replicates for the phosphopeptidome, acetyl peptidome, and glycopeptidome were better clustered than for the proteome analysis. For the phosphopeptidome, variables were more distanced in the LAB sample.

In addition, we generated phosphoproteomic EV data from three distinct breast cancer cell lines corresponding to control (MCF10A), LAB (MCF7), and TN-specific (MDAMB231) breast cancer (manuscript in preparation). By comparing this dataset with our plasma EV data, we identified overlapping phosphoproteins, suggesting that EVs from the plasma can also carry the same subtype-specific phosphoproteins found in EVs from cell lines (**Supplementary Fig. 4, see Supplementary Data 7 for the list of overlapped phosphoproteins**). This finding further highlights the potential of plasma EVs as a non-invasive source for detecting key phosphoproteins associated with LAB and TN breast cancer subtypes.

### Reactome enrichment analysis differentiates LAB and TN BC

**Figure 4.**
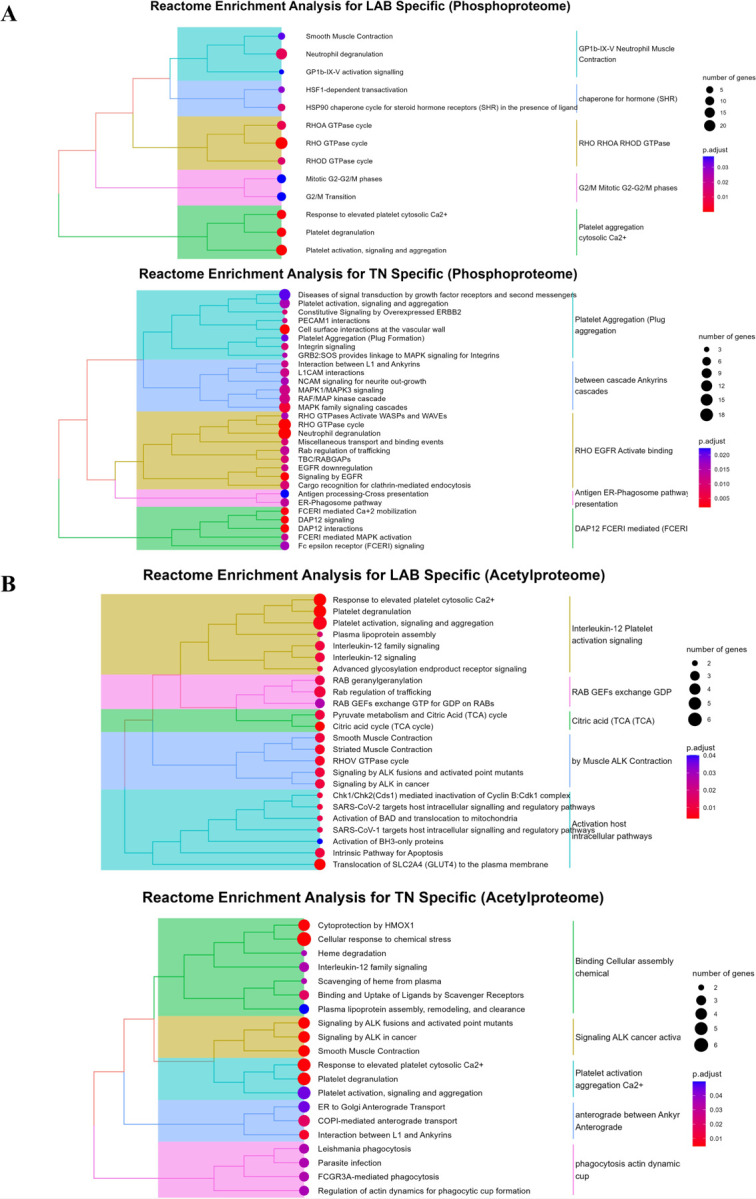
Enrichment analysis of PTM-modified proteins in EVs from breast cancer patients. Reactome pathways enriched in the phosphoproteome (A) and the acetylproteome (B) for luminal A/B and TNBC subtypes.

We analyzed Reactome enrichment analysis of the phosphoproteome, acetylproteome, and glycoproteome for BC subtypes (Figs. 4A–B **and Supplementary Fig. 5, see Supplementary Data 2 for the input lists**). Focusing on Fig. 4A for phosphopeptidome, we can clearly see that many cancer-related signaling pathways, such as EGFR, L1CAM, MAPK, and DAP12, dominated the enriched pathways in TN BC. The interplay of EGFR, L1CAM, MAPK, and DAP12 signaling pathways plays a crucial role in the progression of TN BC, which is characterized by its lack of hormone receptors and HER2 expression, making it particularly aggressive and difficult to treat. In TN BC, the activation of the EGFR pathway often leads to enhanced tumor proliferation and survival, driving the rapid growth that defines this subtype [42–44]. Meanwhile, L1CAM promotes cell adhesion and migration, facilitating invasive behavior and contributing to metastasis, a hallmark of TN BC [45]. Concurrently, the MAPK pathway further drives these processes by enhancing cell division and survival under stressful conditions, which is especially relevant in TN BC, where cellular stress responses are often activated due to aggressive tumor biology. The MAPK pathway, through its regulation of key survival and stress response mechanisms, enables cancer cells to thrive under these conditions, contributing to the tumor’s aggressive growth and resistance to treatment. This makes targeting the MAPK pathway a promising therapeutic strategy for TN BC [46]. Additionally, DAP12 signaling can influence immune evasion, allowing TN BC cells to avoid detection and destruction by the immune system, thus fostering a tumor-promoting microenvironment [47]. Together, these interconnected pathways create a complex network that not only accelerates tumor growth but also contributes to resistance against standard therapies, such as chemotherapy, making TN one of the most challenging BC subtypes to manage and treat effectively. The interplay of EGFR, L1CAM, MAPK, and DAP12 signaling pathways is crucial in TN BC, as their activation can drive tumor proliferation, invasion, and resistance to therapy in this aggressive subtype.

On the other hand, LAB showed the dysregulation of G2/M phases (Fig. 4A). Luminal A cases generally show a more intact G2/M regulation, contributing to a slower proliferation rate and better prognosis. Therapeutic targeting of the G2/M phase is less critical here but could benefit some high-risk patients. Meanwhile, luminal B exhibits higher dysregulation of the G2/M phase, contributing to its more aggressive nature and higher proliferation rates [48]. Therapies targeting G2/M checkpoint proteins (e.g., CDK1, Wee1, CHK1/CHK2) are being actively explored to control tumor growth and improve treatment outcomes.

Unsurprisingly, some pathways are overlapped between LAB and TN, such as the RHO GTPases and platelet aggregation pathways (Fig. 4A). Rho GTPases are key regulators of various cellular processes, including cytoskeletal dynamics, cell migration, proliferation, and survival, all of which are critical in the development and progression of BC [49]. In BC, aberrant activation of these GTPases contributes to enhanced cell motility and invasion, particularly promoting metastasis. This pathway is especially important in more aggressive BC subtypes, such as TN and luminal B, where Rho GTPase signaling promotes metastatic behavior and resistance to therapy. Moreover, platelet aggregation supports BC progression by promoting tumor cell survival, immune evasion, angiogenesis, and metastasis [50].

The acetylproteome analysis revealed distinct pathways compared to those identified in the phosphoproteome, emphasizing the need for examining multiple PTMs to gain a comprehensive understanding of breast cancer subtypes (Fig. 4B). While the phosphoproteome provided insight into certain signaling cascades, the acetylproteome highlighted metabolic and cellular pathways with unique relevance to specific subtypes. For example, as illustrated in Fig. 4B, the acetylproteome analysis of luminal-A and luminal-B subtypes indicated a reliance on the tricarboxylic acid (TCA) cycle for metabolic energy and cellular function, consistent with findings by Lei *et al*. [51]. This reliance on the TCA cycle suggests potential metabolic vulnerabilities that could be explored for targeted therapies. Additionally, the acetylproteome showed shared pathways between LAB and TN, such as platelet activation and ALK-related signaling, which may have implications for disease progression and treatment response.

In addition, surface insulin-like growth factor (IGF) and extracellular matrix (ECM) glycoproteins are specifically upregulated in TN BC (**Supplementary Fig. 5, see Supplementary Data 2 for the input lists**). In TN BC, IGF-1 signaling is crucial for promoting cell proliferation and survival, largely by activating key pathways such as PI3K/AKT and MAPK, which support tumor growth and enable cells to resist apoptosis, underscoring its potential as a therapeutic target [52]. ECM glycoproteins further enhance TN BC progression by modifying the tumor microenvironment; they facilitate critical cell signaling processes, promote tumor cell proliferation, and contribute to the aggressive and invasive characteristics of TN BC, thereby playing a pivotal role in tumor development and metastatic potential [53]. Together, these upregulated components create a pro-tumorigenic environment in TN BC, making IGF-1 and ECM proteoglycans valuable focal points for therapeutic intervention.

To gain a complete understanding of BC biology, it is essential to integrate PTMs on top of the proteome data. While proteome analysis provides valuable insights into the proteins present, the addition of PTMs - such as phosphorylation, acetylation, and glycosylation - reveals dynamic changes in protein function, stability, and interaction that are critical for accurately mapping cellular pathways. By incorporating the PTM data into gene ontology analysis of biological processes, we can achieve a more comprehensive view of pathway regulation and functional protein networks (**Supplementary Fig. 6, see Supplementary Data 2 for the input lists**). Relying on proteome data alone limits the ability to capture these nuanced modifications, thereby restricting pathway analysis and leaving significant gaps in our understanding of BC’s underlying mechanisms.

### The correlation with gene expression in survival analysis

**Figure 5.**
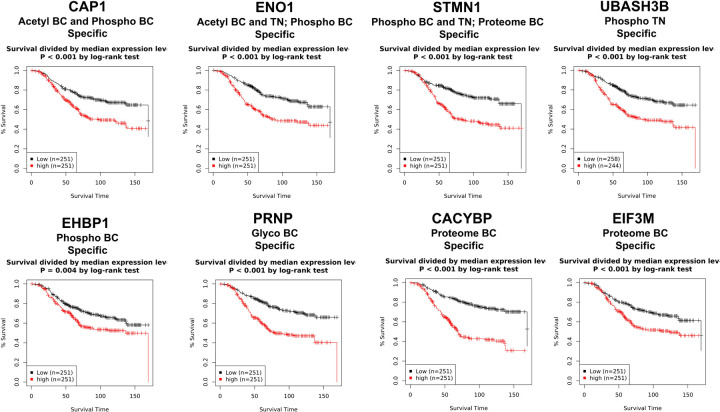
Prognostic markers in extracellular vesicles (EVs) associated with patient survival in breast cancer. This figure highlights key proteins identified as prognostic markers from our EV data, demonstrating their robustness and correlation with patient outcomes. Notably, enolase 1 (EN01), stathmin (STMN1), ubiquitin-associated and SH3 domain-containing B (UBASH3B), calcyclin-binding protein (CACYBP), prion protein (PRNP), EH domain-binding protein 1 (EHBP1), eukaryotic translational initiation factor 3 subunit M (EIF3M), and adenylyl cyclase-associated protein 1 (CAP1) show significant associations with poorer patient survival when overexpressed. These proteins have been extensively discussed in the literature as positive indicators for various breast cancer subtypes.

To demonstrate the robustness of our EV data, we identified several prognostic markers well-supported by existing literature. Notably, Fig. 5 highlights several of the most intriguing features, including adenylyl cyclase-associated protein 1 (CAP1), enolase 1 (ENO1), stathmin, also known as metablastin and oncoprotein-18 (STMN1), ubiquitin associated and SH3 domain-containing B (UBASH3B), EH domain-binding protein 1 (EHBP1), the prion protein (PRNP), calcyclin-binding protein (CACYBP), and eukaryotic translational initiation factor 3 subunit M (EIF3M), which show a significant correlation with poorer patient survival when overexpressed. Previous studies have extensively discussed these markers as positive indicators for various BC subtypes [19, 21]. We observed overexpression of one acetylated peptide of CAP1 and three acetylated peptides of ENO1 in BC, with another acetylated ENO1 peptide specifically elevated in the TN BC subtype. In addition, phosphorylated peptides of CAP1, ENO1, STMN1, and EHBP1 were overexpressed in BC, while phosphorylated STMN1 and UBASH3B peptides showed increased levels in the LAB subtype. The glycosylated peptide of PRNP was also upregulated in BC. Moreover, our data revealed an upregulation of STMN1, CACYBP, and EIF3M proteins across BC cases, while the levels of CAP1, ENO1, UBASH3B, EHBP1, and PRNP showed no significant changes.

ENO1 was previously found to be a positive marker of TN BC [21]. Interestingly, the same phosphorylation site of ENO1 at serine 419 was found to be a tumor-specific marker in TN BC progression [54]. Similar to ENO1, STMN1 protein was also found to be a positive marker of TN BC [21]. STMN1 has been highly associated with aggressive phenotypes of BC [55]. Its overexpression is correlated with BC proliferation in low ER and PR, and high histological grade in human BC [56]. Overall, our study suggests EV markers in plasma are, to some extent, reflective of candidate markers that have been studied in cells or tissues.

### Integrated EV PTMs to distinguish breast cancer subtypes

After the first screening phase was performed with the pooled samples from healthy controls, LAB, and TN BC patients, we selected a group of target-specific markers per modification and BC subtypes. Overall, 135 phosphopeptides, 98 N-glycopeptides, and 47 acetylated peptides were selected for the targeted approach. As shown in Fig. 1, we performed 123 scheduled parallel reaction monitoring (PRM) runs with 41 individual samples (8 controls, 19 LAB, and 14 TN) to quantify individual EV modifications using 700 μL plasma per sample according to the same sequential approach as in the discovery experiment. The PRM data was then exported from Skyline software and analyzed using machine learning, as explained in the methods section and visualized in **Supplementary Fig. 7 (see Supplementary Data 8 for the target lists)**.

**Figure 6.**
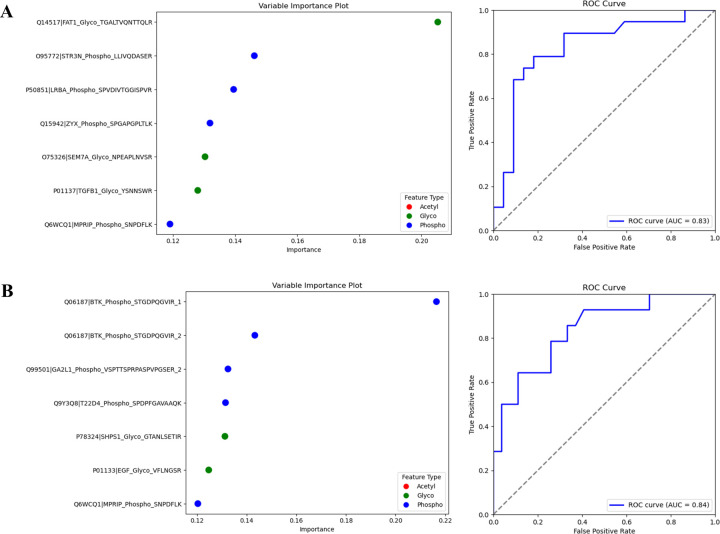
Validation of candidate PTM markers through PRM analysis in individual samples. (A) Variable importance plot (VIP) showing the top features distinguishing luminal A/B (LAB) subtypes from healthy controls and triple-negative breast cancer (TNBC). The corresponding ROC curve analysis demonstrates the diagnostic potential of these PTM markers for this comparison. (B) VIP displaying the top features distinguishing TNBC subtypes from healthy controls and LAB subtypes, with the associated ROC curve analysis highlighting the diagnostic capabilities of the identified PTM markers.

Based on our optimized machine learning algorithm, variable importance classification shows the top seven features distinguishing LAB subtypes from healthy control and TN subtypes: four phosphorylated and three glycosylated targets with an area under the ROC curve (AUC) of 0.83 (Fig. 6A). The top seven targets selected are glycosylated peptides of protocadherin Fat 1 (FAT1), semaphoring 7A (SEM7A), and transforming growth factor beta 1 (TGFB1), as well as the phosphorylated peptides of striatin 3 (STR3N), lipopolysaccharide-responsive beige-like anchor (LRBA), zyxin (ZYX), and myosin phosphatase Rho-interacting protein (MPRIP). The FAT1 protein plays a complex role in BC, acting as both a tumor suppressor and an oncogene. There is not much information about glycosylated FAT1; however, it is important to note that Nantana Kwaepila *et al*. reported high expression levels of FAT1 in BC immunohistochemical analyses of human tumor samples, suggesting that FAT1 expression is expected to be higher [57]. On the contrary, the reduction of FAT1 protein expression has been linked to the progression of BC, aggressive tumor behavior, and unfavorable prognoses [58]. However, the mechanisms connecting the low expression levels of FAT1 in BC to the Wnt/β-catenin signaling pathway remain only partially understood [58]. Our findings may uncover a previously unrecognized avenue for targeting glycosylated FAT1 in BC therapy. Interestingly, Massimo Lodi demonstrated that STR3N, also known as STARD3N, is highly overexpressed in the majority of HER2-positive tumors (including Luminal B) [59]. This finding suggests that STR3N protein expression could be explored as a potential new biomarker in HER2-positive BC.

Meanwhile, variable importance classification shows the top seven features distinguishing TN subtypes from healthy control and LAB subtypes: five phosphorylated and two glycosylated targets with an area under the ROC curve (AUC) of 0.84 (Fig. 6B). The top seven targets selected are phosphorylated peptides of Bruton’s Tyrosine Kinase (BTK), growth arrest-specific 2 like 1 (GA2L1), TSC22 domain family protein 4 (T22D4), and myosin phosphatase Rho-interacting protein (MPRIP), as well as the glycosylated peptides of tyrosine-protein phosphatase non-receptor type (SHPS1) and pro-epidermal growth factor (EGF). BTK has been reported to be expressed in BC cells and to protect these cells from apoptosis [60]. Moreover, there are recently developed inhibitors of BTK, such as ibrutinib (PC1–32765), AVL-292, and CGI-1746, which prevent drug-resistant clones from arising and reduce BC cell survival [61–64].

## DISCUSSION

PTMs play a crucial role in intracellular and intercellular communication by altering protein functions, and irregular PTMs are often linked to the onset and progression of diseases. The identification of various types of PTMs in EVs highlights their important role in mediating intercellular communication. Analyzing PTMs in EVs sheds light on their biogenesis, cargo selection, vesicle uptake and overall function. In this study, we recognize EVs as a unique source for examining the molecular status of original cells with potential clinical implications.

The data generated in this study present a unique opportunity to evaluate and analyze the proteome alongside three distinct PTMs from the same biological samples without compromising data quality (Fig. 1). Using a robust EV PTM enrichment workflow, we detected over 4,600 unique phosphorylation sites, 1,200 N-glyco sites, and 690 acetyl sites from EVs derived from LAB and TN breast cancer subtypes, as well as healthy controls (Fig. 2C). Our findings demonstrate that plasma-derived EVs can serve as surrogates for identifying PTM proteins from breast tumors and detecting specific PTM sites that distinguish between LAB and TN subtypes (Fig. 3). We also demonstrated the importance of evaluating PTMs, as proteomic analysis alone did not yield as much subtype-specific information as PTM analysis. Notably, we observed a significant increase in glycosylation of PD-L1 in BC (Fig. 3C), consistent with recent findings that monoclonal targeting of glycosylated PD-L1 could offer a promising therapeutic approach for TN BC patients [41].

We also generated phosphoproteomic EV data from control (MCF10A), LAB (MCF7), and TN-specific (MDAMB231) breast cancer cell lines (manuscript in preparation) and compared it with plasma EV data. This analysis identified overlapping phosphoproteins (**Supplementary Fig. 4**), suggesting that plasma EVs carry the same subtype-specific phosphoproteins found in cell lines. These shared EV-associated phosphoproteins may serve as valuable biomarkers for disease classification with further investigation.

Furthermore, rather than focusing on novel PTM sites or new functions of PTM proteins, we utilized functional pathway analysis and literature reviews to reveal that many PTM proteins found in plasma EVs have been previously studied in BC tissues, cell lines, or animal models, and are involved in key cancer-related cellular pathways (Fig. 4).

Our EV data analysis identified several prognostic markers, such as ENO1, STMN1, and UBASH3B, which correlate with poorer patient survival and are well-supported by existing literature. Notably, we observed the same phosphorylation site of ENO1 at serine 419, previously reported as a tumor-specific marker in TN BC progression [54], highlighting the potential of plasma EV markers to reflect key candidate markers studied in cells or tissue (Fig. 5). Lastly, we highlight the potential clinical applications by targeting specific subsets of phospho-, N-glyco-, and acetyl-peptides in individual patients, showing their specificity for aggressive BC subtypes. Using bioinformatic integration of targets from three PTMs, we developed a panel of seven candidate markers capable of distinguishing BC subtypes (Fig. 6). Among these, BTK, FAT1, and STR3N were significantly upregulated and have previously been linked to BC progression in cell culture systems and tissue samples. With EVs showing great potential as a source of circulating biomarkers, our findings open new avenues for diagnostic tools that could inform and improve therapeutic decision-making in BC treatment.

Overall, our study highlights the potential of EVs and PTMs found in EVs for disease diagnostics, particularly BC. A key challenge, however, lies in translating these identified candidate markers into large-scale prospective studies for validation and eventual clinical application - a hurdle common to liquid biopsy approaches. Despite this, our findings offer crucial insights into platforms that could improve therapeutic strategies. Moving forward, substantial efforts will be required to develop EV-based clinical assays that deliver real clinical benefits and can be seamlessly integrated into routine clinical practice.

## Supplementary Material

Supplementary Files

This is a list of supplementary files associated with this preprint. Click to download.

- SupplementaryData.zip
- Slide9.jpg
- Slide10.jpg
- Slide11.jpg
- Slide12.jpg
- Slide13.jpg
- Slide14.jpg
- Slide15.jpg
- Slide16.jpg
- Slide17.jpg

## Data Availability

The mass spectrometry raw data files and Proteome Discoverer search results have been deposited in the jPOSTrepo database (https://repository.jpostdb.org/preview/161878879467242b62ae7a7) [65]. The dataset identifier is JPST003449 | PXD057412. The reviewers can access them with an access key 2314. The PRM raw data files and the Skyline file have been deposited in the Panorama Public (https://panoramaweb.org/bc.url). The ProteomeXchange ID reserved for these data is: PXD057449 (https://proteomecentral.proteomexchange.org/cgi/GetDataset?ID=PXD057449). The DOI for the data is: https://doi.org/10.6069/js1s-k435. Here are the reviewer account details: Email: panorama+reviewer299@proteinms.net Password: 9=83jcs7O%@pBA

## Code Availability

Custom Python code used for feature selections, model training, and predictive analyses, along with the README file, input files, and expected results, have been deposited in GitFront (https://gitfront.io/r/rvchit/DQCiPixbEQTt/Tao-Lab-Breast-Cancer/).
